# Extracorporeal Shockwave Therapy for Diabetes Related Foot Ulcers: A Pilot Three‐Arm Double‐Blinded Randomised Controlled Trial

**DOI:** 10.1111/iwj.70740

**Published:** 2025-11-30

**Authors:** L. Hitchman, R. Lathan, B. Ravindhran, M. Sidapra, J. Long, A. Cowling, A. Keding, J. Watson, C. Iglesias, G. Smith, M. Twiddy, D. Russell, I. C. Chetter

**Affiliations:** ^1^ Faculty of Clinical Sciences, Hull York Medical School Heslington UK; ^2^ Hull University Teaching Hospitals NHS Trust Heslington UK; ^3^ York Trials Unit, Department of Health Sciences University of York York UK; ^4^ Department of Health Sciences University of York York UK; ^5^ Institute of Clinical and Applied Sciences, Hull York Medical School Heslington UK; ^6^ Leeds Vascular Institute, Leeds Teaching Hospitals NHS Trust Leeds UK; ^7^ Leeds Institute of Clinical Trials Research, University of Leeds Leeds UK

**Keywords:** diabetic foot ulcers, extracorporeal shockwave therapy, feasibility studies, randomised controlled trial, wound healing

## Abstract

There is an urgent need for effective interventions to aid diabetes‐related foot ulcer (DFU) healing. This study aimed to test the deliverability of a proposed trial of extracorporeal shockwave therapy (ESWT) for DFU healing. A pilot double‐blinded randomised controlled trial. Patients with a DFU present for ≥ 4 weeks were randomised to high dose (500 shocks/cm^2^), low dose (100 shocks/cm^2^) or sham (0 shocks/cm^2^) ESWT, plus standard care. Follow‐up was for 24 weeks. Primary outcome was deliverability of the trial. Secondary outcomes were healing, quality of life and healthcare resource use. One‐hundred and forty‐one (15.6%) screened patients were eligible and 74 (52.5%) patients were recruited. Follow‐up attendance was 97.3% (72/74), 93.2% (69/74) and 87.8% (65/74) at 6, 12 and 24 weeks. The median DFU healing time was high dose: 54.0 (IQR 119.0), low dose: 78.5 (IQR 61.0) and sham: 83.0 (IQR 85.0) days. The mean EQ‐5D‐5L utility value at 24 weeks was high dose: 0.621 (95% CI 0.438–0.804), low dose: 0.779 (95% CI 0.683–0.876) and sham: 0.806 (95% CI 0.717–0.895). Healthcare resource use was lowest in the low‐dose ESWT arm. The pilot trial has demonstrated that patients with a DFU are willing to engage in the proposed trial and suggest the optimal way to deliver the definitive trial.

**Summary**

- A definitive trial of ESWT for DFU can expect around 15% of screened patients attending podiatry services with a DFU to be eligible and around 50% of eligible patients to enter the trial.
- Participant adherence to the trial arm allocated treatment is likely to be high.
- The trial attrition at 6 months following randomisation is expected to be low.
- High‐dose ESWT may be associated with improved healing at 6 months compared to low dose ESWT and standard care alone.
- A fully powered multi‐centre randomised controlled trial is deliverable and required to definitively explore the efficacy of ESWT for DFU healing.

## 1 Background

Diabetes related foot ulcers (DFU) have a huge impact on patients, healthcare and society. It is estimated that 537 million people are living with diabetes, of which between 12% and 34% will develop a DFU [1, 2, 3]. Prolonged healing of DFUs, exacerbated by infection, ischaemia and pressure, predisposes patients to multiple hospital admissions, amputations and increased mortality [4, 5, 6]. People living with DFUs have significant disability and consequent severe quality‐of‐life impairment [5, 7, 8]. The treatment of DFUs is costly to patients, healthcare systems and society. The estimated cost of treating DFUs to the National Health Service in England is nearly £1 billion per year [7]. In the United States of America, the cost is between $11 and $17 billion [9]. This does not include costs incurred by patients nor the loss of productivity to society.

Advances in DFU care struggle to translate into improved outcomes [10, 11]. Guideline recommendations are often underpinned by low certainty evidence or are best practice statements [12, 13, 14, 15]. This highlights the urgent need to investigate which interventions are effective in improving DFU‐related outcomes. The routine use of new interventions that lack a high‐quality evidence base risks care being potentially ineffective and valuable resource wastage.

Extracorporeal shockwave therapy (ESWT) is a non‐invasive intervention which uses high‐frequency soundwaves and is hypothesised to improve healing by stimulating angiogenesis and epithelisation and regulating inflammation [16, 17, 18, 19]. Developing evidence has demonstrated the potential effectiveness in healing DFUs; however, there is uncertainty due to biases in existing study designs and execution [20]. In addition, the existing studies have used different doses of ESWT. This makes it challenging to integrate ESWT into routine care, if it is proven to be effective and difficult to combine studies to estimate an effect size.

This pilot three‐arm double‐blinded randomised controlled trial (RCT) aimed to explore the deliverability of a definitive trial investigating the clinical efficacy of ESWT for DFU healing. This pilot trial was conducted to ensure the trial design is robust and will be able to address the research question.

## 2 Methods

The trial is reported with reference to the Consolidated Standards of Reporting Trials (CONSORT) 2010 statement: extension to randomised pilot and feasibility studies [21].

### 2.1 Design

A single centre pilot three‐arm double‐blinded RCT of high dose, low dose and sham ESWT for DFU healing. The trial was conducted in the outpatient setting in a tertiary care hospital in England.

### 2.2 Participants

Patients with a DFU present for 4 weeks or longer, sufficient perfusion and capacity to consent and engage in the trial processes were eligible (Table 1). Patients with DFU soft tissue infection, without osteomyelitis and surgical wounds healing by secondary intention (SWHSI) in people living with diabetes, present for 4 weeks or longer, were also eligible for inclusion. Patients with a DFU less than 4 weeks old were screened again when the DFU was 4 weeks old. Initially, patients who were taking anti‐coagulation were excluded. This criterion was removed following review of the guidelines and discussion with the manufacturer after 11 months of recruitment [22]. Patients were screened from the outpatient podiatry and multidisciplinary diabetic foot clinics, community podiatry clinics and inpatient settings.

**TABLE 1:** Eligibility criteria.

Inclusion criteria	Exclusion criteria
DFU present for ≥ 4 weeks	Interdigital DFU (as the ESWT paddle does not fit between the digits)
Absolute toe pressure ≥ 50 mmHg or an ankle brachial pressure index ≥ 0.7	Diagnosis of malignancy in the treatment area or disseminated, haematological or lymphatic malignancy (contraindication to ESWT)
Capacity to consent	Pregnant, trying to conceive or breast feeding (contraindication to ESWT)
Willing to receive any of the trial interventions	Active participants in other wound/DFU‐related trial
Willing to have their DFU photographed	Osteomyelitis^a^
Willing to comply with the follow‐up schedule	Anticoagulation^b^

Abbreviation: DFU: diabetic foot ulcer.

^a^ Clinical suspicion, receiving treatment or proven (radiological or microbiology sample) osteomyelitis.

^b^ Removed after 11 months of recruitment.

### 2.3 Interventions

ESWT was delivered using the PiezoWave2 device (Richard Wolfe, Germany) in the outpatient setting. The DFU was undressed, sharp debrided if necessary and covered in a clear film. Ultrasound gel was used for coupling between the gel paddle and DFU bed, with care to remove any air bubbles.

Focused volume shockwaves were delivered at 0.1 mJ/mm^2^ at 5 Hz and penetrated 5 mm. In the low dose arm, participants received 100 shocks/cm^2^ (minimum dose 100 shocks) and in the high dose arm, participants received 500 shocks/cm^2^ (minimum dose 500 shocks). In the sham arm, participants did not receive any shockwaves; instead, the ESWT machine and DFU were prepared in the same way and a 6‐min audio recording of ESWT was played. Participants received the intervention three times over a 7 ± 2 day period.

In addition, all patients received standard DFU care as outlined in the National Institute of Clinical Excellence (NICE) and International Working Group on the Diabetic Foot (IWGDF) guidelines [12, 13, 14, 15, 23].

### 2.4 Outcomes

The primary outcome was deliverability of the trial. This included: the percentage of patients who were eligible, the percentage of eligible patients recruited, adherence to treatment, adherence to follow‐up and amount of missing data. Data was collected at each follow‐up point and overall. The stop/go criteria used to judge deliverability were [24]:

– Green: > 60% of eligible patients recruited, adherence to treatment arm and attendance to follow‐up
– Amber: 30%–60% of eligible patients recruited, adherence to treatment arm and attendance to follow‐up
– Red: < 30% of eligible patients recruited, adherence to treatment arm and attendance to follow‐up

Secondary outcomes included: time to healing, proportion of DFU healed, change in DFU size, quality of life, adverse events and healthcare resource use. Healing was defined as complete epithelialisation, without eschar, present for at least 2 weeks. Healing was reported by the patient or a clinician. The patient was then reviewed by the research team within 3 days to confirm healing and again at 2 weeks to ensure the DFU remained healed. DFUs were measured using a tape measure and probe on the longest axis of the length, width and depth.

Quality of life was measured using EQ‐5D‐5L [25], Diabetic Foot Ulcer Scale Short Form (DFS‐SF) [26] and Wound‐Qol‐14 [27, 28, 29]. EQ‐5D‐5L is a generic quality‐of‐life tool and measures quality of life across five domains to generate a single score (utility value). The utility values were derived from the English value set [30, 31]. EQ‐5D‐5L also includes a visual analogue scale (VAS) for the patient to rate how good or bad their health is that day between 0 (worst health imaginable) and 100 (best health imaginable). A higher utility value and VAS score indicate a better quality of life. DFS‐SF measures disease‐specific quality of life across six domains. A higher score indicates a better quality of life. Wound‐Qol‐14 measures disease‐specific quality of life over three domains and generates a global score. A lower score indicates a better quality of life.

Healthcare resource use related to DFU care was recorded in a patient healthcare diary and cross checked with electronic patient records. Secondary outcomes (excluding time to healing) were collected after the third ESWT session and at 6, 12 and 24 weeks after randomisation in the outpatient or in‐patient setting.

### 2.5 Sample Size

The target sample size was 90 participants. This was based on the minimum number of participants to estimate the standard deviation to base a sample size calculation on for a definitive trial [32, 33, 34, 35, 36], with 30% attrition.

### 2.6 Randomisation

Stratified block randomisation, using randomly varying block sizes of 3 and 6, stratifying participants by ulcer size (≤ 1 or > 1 cm^2^) and aetiology (neuropathic DFU or neuroischaemic DFU) was used. Neuropathic DFU was defined as an ABPI > 0.8 or absolute toe pressure > 60 mmg. Neuroischaemic DFU was defined as an ABPI between 0.70–0.79 or an absolute toe pressure between 50 and 59 mmHg. The online randomisation tool was developed by an independent statistician at York Trials Unit. The research team did not have access to the allocation sequence.

### 2.7 Blinding

Trial participants were blinded to the treatment allocation. ESWT is not routinely used in standard DFU care, so participants were unlikely to be able to identify the active arms. Clinicians assessing whether the DFU was healed were blinded to participants' trial arm allocation.

### 2.8 Statistical Analysis

Descriptive statistics using counts, percentages, means, standard deviations, medians and interquartile ranges were used to summarise the results. Between‐group differences are presented with confidence intervals. Change in DFU area is presented as median percentage change in DFU size at each time point. Statistical testing was not performed as this is a pilot RCT and is not powered to detect between‐group differences. The effect size for quality‐of‐life measurements was calculated by subtracting the median score at baseline from the median score and dividing by the interquartile range at each follow‐up point [37]. An effect size ≥ 0.5 signifies a difference. The data was analysed using intention‐to‐treat principles.

Analysis was performed using IMB SPSS (Version 28.0.0; Armonk, NY, USA) and STATA (Version 18; TX, USA).

## 3 Results

**FIGURE 1:**
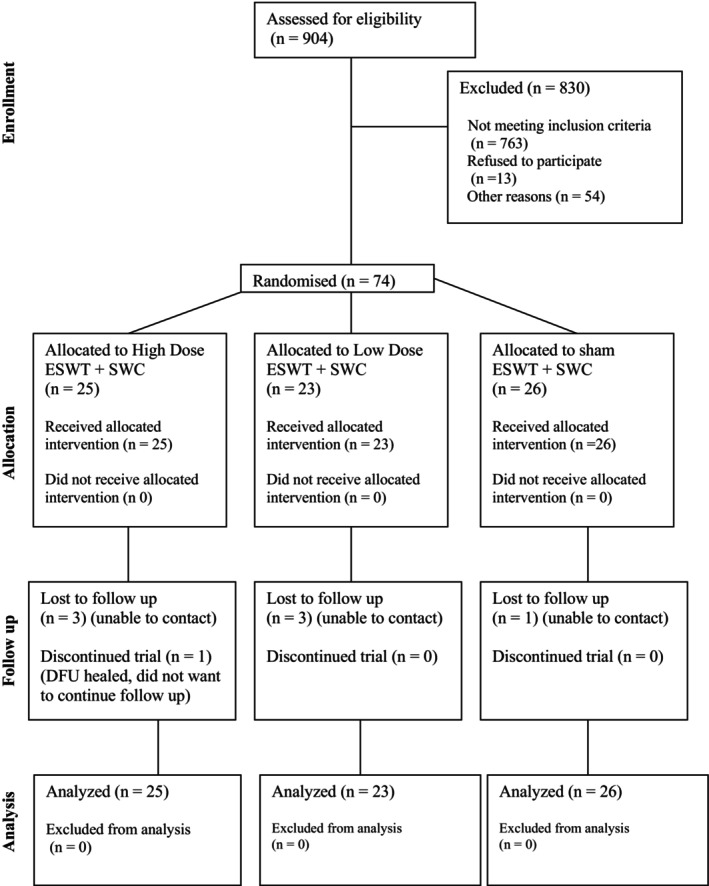
Consort flow diagram. ESWT: extracorporeal shockwave therapy; SWC: standard wound care.

The pilot trial recruited 74 participants between 26 May 2022 and 31 October 2023 (Figure 1). The majority of participants were males with Type 2 Diabetes Mellitus (Table 2). The mean age of participants was 61.5 (SD 11.2) years. The forefoot was the most common location of the DFU. The mean DFU area was 6.9cm^2^ (SD 11.5) and the mean DFU age was 6.1 months (SD 9.1) (Table 3).

**TABLE 2:** Participant demographics.

Demographic	High‐dose ESWT (*N* = 25)	Low‐dose ESWT (*N* = 23)	Sham ESWT (*N* = 26)
Age, years, mean (SD)	56.6 (11.2)	61.4 (8.5)	66.3 (11.5)
Sex, *n* (%)			
Male	19 (76.0)	17 (73.9)	24 (92.3)
Female	6 (14.0)	6 (26.1)	2 (7.7)
Ethnicity, *n* (%)			
White British	23 (92.0)	23 (100)	26 (100)
South Asian	2 (8.0)	0	0
Smoking, *n* (%)			
Current	3 (12.0)	4 (17.4)	4 (15.4)
Ex‐smoker	9 (36.0)	8 (34.8)	14 (53.8)
Non‐smoker	13 (52.0)	11 (47.8)	8 (30.8)
Alcoholic intake, units/week, mean (SD)	6.0 (11.6)	3.1 (5.6)	13.4 (23.5)
BMI, kg/m^2^, mean (SD)	30.7 (6.3)	33.5 (6.8)	30.1 (5.5)
Type of diabetes, *n* (%)			
Type 1	2 (8.0)	3 (13.0)	3 (11.5)
Type 2	23 (92.0)	20 (87.0)	23 (88.5)
Duration of diabetes, months, mean (SD)	135.0 (100.9)	241.9 (132.3)	200.1 (105.6)
HbA1c, mmol/mol, mean (SD)	73.7 (27.6)	80.2 (26.4)	72.3 (23.8)
Diabetes management, *n* (%)			
Diet controlled	2 (8.0)	2 (8.7)	0
Oral anti‐hyperglycaemics	18 (72.0)	13 (56.5)	20 (76.9)
Injec anti‐hyperglycaemics	1 (4.0)	0	1 (3.8)
Insulin	11 (44%)	15 (65.2)	15 (57.7)
Comorbidities, *n* (%)			
COPD	1 (4.0)	0	1 (3.8)
CKD	9 (36.0)	5 (21.7)	10 (38.5)
IHD	4 (16.0)	4 (17.4)	6 (23.1)
Heart failure	1 (4.0)	1 (4.3)	3 (11.5)
HTN	13 (52.0)	14 (60.9)	16 (61.5)
Stroke	2 (8.0)	0	2 (7.7)
TIA	3 (12.0)	0	1 (3.8)
PAD	1 (4.0)	3 (8.7)	3 (11.5)
Index limb revascularisation	2 (8.0)^a^	3 (8.7)	4 (15.4)^b^
Index limb TMA	3 (12.0)	1 (4.3)	0
Index limb minor	9 (36.0)	9 (39.1)	4 (15.4)
Amputation/debridement			
Contralateral limb revascularisation	0	0	0
Contralateral limb MLLA	0	0	1 (3.8)
Contralateral limb TMA	1 (4.0)	1 (4.3)	0
Contralateral limb minor amputation/debridement	3 (12.0)	3 (13.0)	1 (3.8)
Secondary prevention, *n* (%)			
Anti‐platelet	7 (28.0)	8 (34.8)	6 (23.1)
Statin	14 (56.0)	16 (69.6)	23 (88.5)
Medication use, *n* (%)			
Prednisolone	1 (4.0)	0	0
Iron replacement	3 (12.0)	7 (30.4)	4 (15.4)

Abbreviations: BMI: body mass index; CKD: chronic kidney disease; COPD: chronic obstructive pulmonary disease; HTN: hypertension; IHD: ischaemia heart disease; MLLA: major lower limb amputation; PAD: peripheral arterial disease; SD: standard deviation; TIA: transient ischaemic attack; TMA: trans‐metatarsal amputation.

^a^ Revascularisation for trauma.

^b^ Revascularisation during cancer excision.

**TABLE 3:** DFU CHARACTERISTICS.

Ulcer characteristics	High‐dose ESWT (*N* = 25), *n* (%)	Low‐dose ESWT (*N* = 23), *n* (%)	Sham ESWT (*N* = 26), *n* (%)
SWHSI	5 (20.0)	3 (13.0)	1 (3.8)
Neuro‐ischaemic	3 (12.0)	3 (13.0)	4 (15.4)
Previous DFU, *n* (%)	16 (64.0)	16 (69.6)	14 (53.8)
Ulcer site, *n* (%)			
Forefoot	13 (52.0)	17 (73.9)	18 (69.2)
Midfoot	6 (24.0)	3 (13.0)	4 (15.4)
Hindfoot	6 (24.0)	3 (13.0)	4 (15.4)
Ulcer laterality, *n* (%)			
Left	11 (44.0)	12 (52.2)	11 (42.3)
Right	14 (56)	11 (47.8)	15 (57.7)
DFU age, mean (SD), days	133.5 (167.5)	239.7 (411.6)	180.6 (184.2)
DFU area, mean (SD), cm^2^	9.5 (14.8)	6.6 (12.1)	4.6 (5.5)
DFU depth, mean, (SD), cm	0.4 (0.6)	0.2 (0.2)	0.3 (0.2)
SINBAD Score, *n* (%)			
1	2 (8.0)	3 (13.0)	5 (19.2)
2	11 (44.0)	11 (47.8)	7 (26.9)
3	9 (36.0)	6 (26.1)	9 (34.6)
4	2 (8.0)	2 (8.7)	4 (15.4)
5	1 (4.0)	1 (4.4)	1 (3.9)
6	0	0	0
WIfI stage, *n* (%)			
1	15 (60.0)	20 (87.0)	18 (69.2)
2	8 (32.0)	3 (13.0)	8 (30.8)
3	1 (4.0)	0	0
4	1 (4.0)	0	0
ABPI, mean (SD)	1.2 (0.3)	1.1 (0.2)	1.3 (0.2)
TBPI, mean (SD)	0.9 (0.3)	0.9 (0.3)	0.8 (0.3)
Foot deformity, *n* (%)			
Claw toes	1 (4.0)	4 (17.4)	4 (15.4)
Hammer toes	2 (8.0)	4 (17.4)	6 (23.1)
Rocker bottom	5 (30.0)	5 (21.7)	5 (19.2)
Prominent MTH	1 (4.0)	0	0
Offloading device at baseline			
Removable BA cast	3 (12.0)	1 (4.4)	4 (15.4)
Removable BK offloading device	1 (4.0)	0	0
Offloading sandals (forefoot/hindfoot)	10 (40.0)	11 (47.8)	9 (34.6)
Custom footwear	2 (8.0)	5 (21.7)	6 (23.1)
Total contact insole	4 (16.0)	4 (17.4)	6 (23.1)
Over the counter insole	0	0	1 (3.9)
Felt padding	6 (24.0)	6 (26.1)	5 (19.2)
Wheelchair	2 (8.0)	2 (8.7)	1 (3.9)
Gait aid	5 (20.0)	1 (4.4)	5 (19.2)
Foam heel cup	1 (4.0)	1 (4.4)	0

Abbreviations: ABPI: ankle brachial pressure index; BA: below ankle; BK: below knee; DFU: diabetic foot ulcer; SD: standard deviation; SINBAD: site, ischaemia, neuropathy, bacterial infection, area, depth; SWHSI: surgical wound healing by secondary intention; TBPI: toe brachial pressure index; TH: metatarsal head; WIfI: wound, ischaemia, foot infection.

### 3.1 Pilot Outcomes

Nine hundred and four patients were screened for eligibility during the 18 months recruitment period. 141 (15.6%) patients met the eligibility criteria. The most frequent reasons that patients were not eligible were: DFU less than 4 weeks old and either healed or met another exclusion criterion on re‐screening (274/763; 35.9%), ABPI < 0.7 or absolute toe pressure < 50 mmHg (108/763; 14.2%) and osteomyelitis (108/763; 14.2%) (Table A1). Of the 141 eligible patients, 74 (52.5%) were recruited to the trial. Reasons eligible patients were not recruited included being unable to contact the patient following the screening visit (14/67; 20.9%), patient declined participation without providing a reason (13/67; 19.4%) and patient reported they could not find suitable transport to attend the ESWT sessions (8/67; 11.9%) (Table A2).

All participants attended the first and third intervention appointments. One participant in the high‐dose arm did not attend their second appointment. Twenty‐four (96.0%; 24/25) participants in the high dose arm received three sessions, all (100%; 23/23) participants in the low dose arm received all three intervention sessions and 24 (92.3%) participants in the sham arm received all three sessions. Reasons for ESWT not being delivered included missing the appointment (high dose arm *n* = 1), too unwell on presentation (high dose arm *n* = 1, same patient), attended out of the treatment window (sham arm = 1) and isolated on an infection ward (sham arm *n* = 1). The overall percentage of participants who attended ESWT sessions and received their allocated treatment was 94.6% (70/74). The standard of care delivered is reported in the Table A3, A4, A5, A6.

**FIGURE 2:**
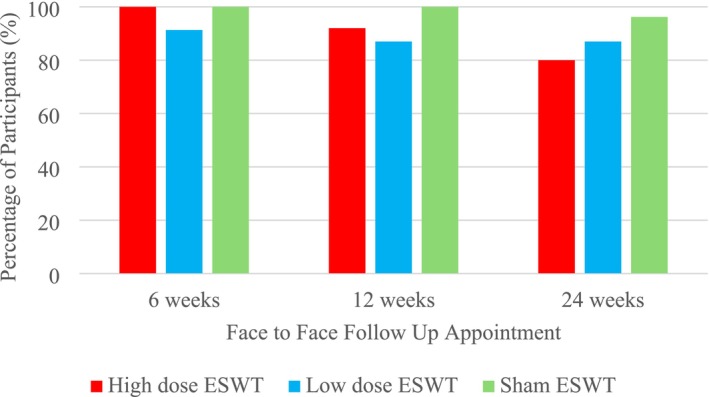
Differences in follow‐up attendance between the trial arms.

Attendance at the 6‐, 12‐ and 24‐week follow‐up appointments was 97.3% (72/74), 93.2% (69/74) and 87.8% (64/74). A higher proportion of participants in the sham arm attended follow‐up appointments, compared to the low dose and high dose arms (Figure 2).

Data completeness for ulcer measurements, 2D photograph, quality of life questionnaires, WIfI stage and SINBAD score were assessed. The most frequently incomplete data items were the quality‐of‐life questionnaires (Table 4). Completion of the disease‐specific quality‐of‐life tools was slightly less than EQ‐5D‐5L. Wound measurements, DFU classification and 2D photographs were less frequently missing than the quality‐of‐life measurements.

**TABLE 4:** Missing data.

Data item	High‐dose ESWT (*N* = 25)	Low‐dose ESWT (*N* = 23)	Sham ESWT (*N* = 26)	Total missing (*N* = 74)
*6 weeks follow‐up*				
Ulcer Measurements,^a^ *n* (%)	1 (4.0)	2 (8.7)	0	3 (4.1)
2D photograph,^a^ *n* (%)	0	0	0	0
Quality of life questionnaires, *n* (%)				
DFS‐SF	3 (12.0)	2 (8.7)	1 (3.8)	6 (8.1)
Wound‐Qol‐14	3 (12.0)	2 (8.7)	1 (3.8)	6 (8.1)
EQ‐5D‐5L	2 (8.0)	2 (8.7)	1 (3.8)	6 (8.1)
WIfI stage, *n* (%)	1 (4.0)	2 (8.7)	0	3 (4.1)
SINBAD score, *n* (%)	0	0	0	0
*12 weeks follow‐up*				
Ulcer measurements,^a^ *n* (%)	1 (4.0)	1 (4.3)	0	2 (2.7)
2D photograph,^a^ *n* (%)	0	0	0	0
Quality of life questionnaires, *n* (%)				
DFS‐SF	2 (8.0)	4 (17.4)	0	6 (8.1)
Wound‐Qol‐14	2 (8.0)	4 (17.4)	0	6 (8.1)
EQ‐5D‐5L	2 (8.0)	4 (17.4)	0	6 (8.1)
WIfI stage, *n* (%)	1 (4.0)	1 (4.3)	0	0
SINBAD score, *n* (%)	0	0	0	0
*24 weeks follow‐up*				
Ulcer measurements,^a^ *n* (%)	3 (12.0)	3 (13.0)	1 (3.8)	7 (9.5)
2D photograph,^a^ *n* (%)	1 (4.0)	0	2 (7.7)	3 (4.1)
Quality of life questionnaires, *n* (%)				
DFS‐SF	5 (20.0)	4 (17.4)	2 (7.7)	11 (14.9)
Wound‐Qol‐14	5 (20.0)	4 (17.4)	2 (7.7)	11 (14.9)
EQ‐5D‐5L	4 (16.0)	4 (17.4)	1 (3.8)	9 (12.2)
WIfI stage, *n* (%)	2 (8.0)	3 (13.0)	1 (3.8)	6 (8.1)
SINBAD score, *n* (%)	0	0	0	0

^a^ Out of total attendees unhealed at that timepoint.

### 3.2 Clinical Outcomes

#### 3.2.1 Proportion of DFU Healed

By 6 weeks, 36.0% (9/25) of DFUs healed in the high dose ESWT arm, 17.4% (4/23) healed in the low dose arm and 11.5% (3/26) healed in the sham arm. At 12 weeks, 40% (10/25) of DFUs healed in the high dose ESWT arm, 39.1% (9/23) healed in the low dose arm and 23% (6/26) healed in the sham arm. At 24 weeks, 64.0% (16/25) of DFUs healed in the high dose ESWT arm, 56.5% (13/23) healed in the low dose arm and 42.3% (11/26) healed in the sham arm.

#### 3.2.2 Change in DFU Size

**FIGURE 3:**
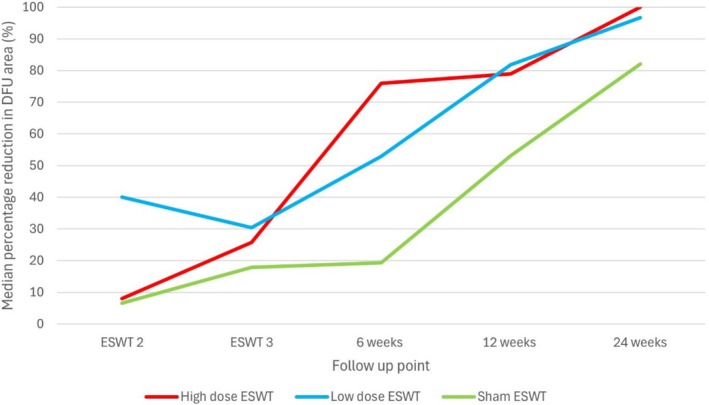
Change in DFU size during the pilot trial.

Between baseline and the 6‐week follow‐up, the median percentage reduction in DFU area was 75.9% (IQR 54.2) in the high dose ESWT arm, 52.9% (IQR 116.8) in the low dose ESWT arm and 19.3% (IQR 84.0) in the sham ESWT arm. At the 12‐week follow‐up, the median percentage reduction in DFU area was 78.9% (IQR 76.8) in the high dose arm, 81.8% (IQR 182.5) in the low dose arm and 53.1% (IQR 148.3) in the sham arm. The median percentage reduction in DFU size between baseline and 24 weeks was 100% (IQR 3.8) in the high dose arm, 96.7% (IQR 91.8) in the low dose arm and 82.0% (IQR 63.5) in the sham arm (Figure 3).

#### 3.2.3 Time to DFU Healing

**FIGURE 4:**
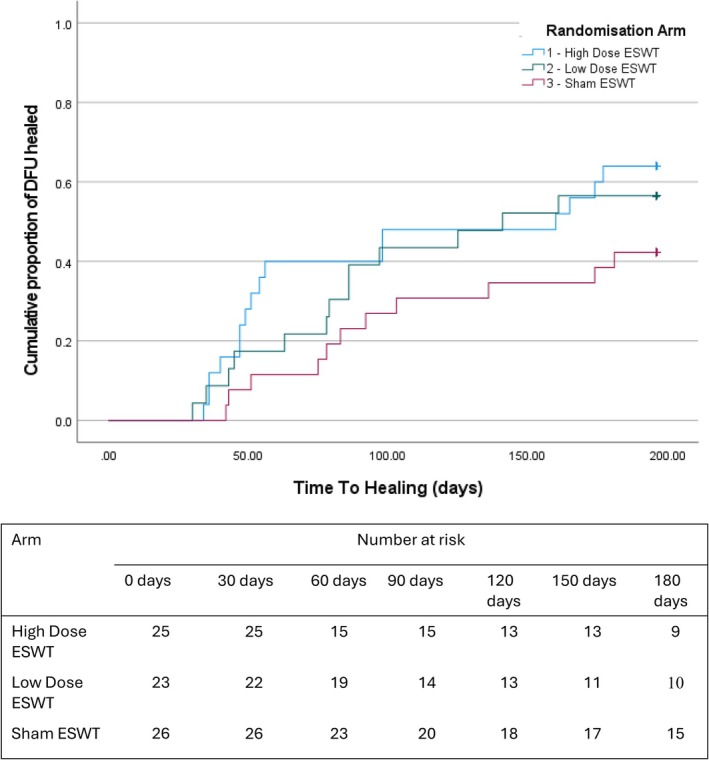
Kaplan–Meier graph of time to DFU healing.

In the participants whose DFU healed during the 6‐month follow‐up, the median time to healing was 54.0 (IQR 119.0) days in the high dose ESWT arm, 78.5 (IQR 61.0) days in the low dose arm and 83.0 (IQR 85.0) days in the sham arm (Figure 4).

### 3.3 Health‐Related Quality of Life

#### 3.3.1 EQ‐5D‐5L

The mean utility value in the high‐dose ESWT arm (*n* = 25) was 0.604 (95% CI 0.439–0.769) at baseline compared to 0.621 (95% CI 0.438–0.804) at 24 weeks. In the low‐dose arm (*n* = 23) the mean utility value was 0.660 (95% CI 0.528–0.791) at baseline compared to 0.779 (95% CI 0.683–0.876) at 24 weeks. In the sham arm (*n* = 26), the mean utility value was 0.781 (95% CI 0.709–0.858) at baseline compared to 0.806 (95% CI 0.717–0.895) at 24 weeks. The largest improvement in utility value was in the low‐dose arm (effect size 0.34) (Table A7).

In the high‐dose ESWT arm, the mean EQ‐5D‐5L VAS score, rating how good or bad the patient thought their health was that day, was 61.0 (95% CI 49.3–72.6) at baseline compared to 60.8 (95% CI 46.5–75.1) at 24 weeks. In the low dose arm, the mean EQ‐5D‐5L VAS score was 63.3 (95% 51.2–75.3) at baseline and 63.6 (95% CI 49.0–78.2) at 24 weeks. In the sham arm, the mean VAS score was 67.2 (95% CI 56.8–77.6) at baseline and 74.5 (95% CI 66.3–82.7) at 24 weeks. The largest improvement in VAS score was in the high dose arm (effect size 0.20) (Table A7).

#### 3.3.2 Diabetic Foot Ulcer Scale—Short Form

##### 3.3.2.1 Leisure

The mean leisure score in the high‐dose ESWT arm (*n* = 25) at baseline was 65.8 (95% CI 50.6–81.0) compared to 80.3 (95% CI 64.7–95.8) at 24 weeks. In the low dose arm (*n* = 23), the mean score was 55.6 (95% CI 37.1–74.1) at baseline and 77.1 (95% CI 61.1–93.0) at 24 weeks. In the sham arm (*n* = 26) at baseline, the mean score was 47.9 (95% CI 33.9–61.8) and increased to 71.2 (95% CI 56.3–86.1) at 24 weeks. The high‐dose arm had the greatest improvement in leisure score (effect size 0.78) (Table A8).

##### 3.3.2.2 Physical Health Domain

The mean physical health score in the high‐dose ESWT arm at baseline was 68.9 (95% CI 53.7–84.2) and 76.6 (95% CI 62.6–90.6) at 24 weeks. In the low‐dose arm, the mean physical health score was 74.4 (95% CI 60.0–88.8) at baseline and 82.4 (95% CI 72.6–92.7) at 24 weeks. In the sham arm, the mean physical health score was 65.5 (95% CI 51.8–79.2) at baseline and 78.1 (95% CI 66.1–90.1) at 24 weeks. The high‐dose arm had the greatest improvement in physical health score (effect size 0.51) (Table A8).

##### 3.3.2.3 Dependence/Daily Life

The mean dependence/daily life score in the high dose ESWT arm at baseline was 83.7 (95% CI 73.2–94.1) and 88.9 (95% CI 79.4–98.5) at 24 weeks. In the low‐dose arm, the mean score was 64.4 (95% CI 50.1–78.7) at baseline and 85.6 (95% CI 75.1–96.1) at 24 weeks. In the sham arm, the mean was 71.9 (95% CI 62.1–81.7) at baseline and 80.0 (95% CI 68.9–91.1) at 24 weeks. The greatest improvement was in the low dose arm (effect size 0.50) (Table A8).

##### 3.3.2.4 Negative Emotions

The mean negative emotions score in the high‐dose ESWT arm at baseline was 76.5 (95% CI 65.0–88.0) and 78.9 (95% CI 65.2–92.7) at 24 weeks. The mean score in the low‐dose arm at baseline was 62.7 (95% CI 47.7–77.8) and 75.7 (95% CI 58.8–92.7) at 24 weeks. In the sham arm, the mean score was 55.0 (95% CI 38.0–72.0) at baseline and 71.4 (95% CI 57.6–85.3) at 24 weeks. The greatest improvement was in the low‐dose arm (effect size 0.40) (Table A8).

##### 3.3.2.5 Worried About Ulcers/Feet

The mean worried about ulcers/feet score in the high dose ESWT arm at baseline was 54.9 (95% CI 38.6–71.3) and 73.0 (95% CI 56.6–89.4) at 24 weeks. In the low‐dose arm, the mean score was 47.4 (95% CI 26.3–68.3) at baseline and 71.3 (95% CI 55.0–87.6) at 24 weeks. In the sham arm, the mean score was 47.6 (95% CI 30.2–65.1) at baseline and 60.7 (95% CI 44.3–77.1) at 24 weeks. The greatest improvement in score was in the high dose arm (effect size 0.48) (Table A8).

##### 3.3.2.6 Bothered by Ulcer Care

The mean bothered by ulcer care score in the high‐dose ESWT arm at baseline was 69.4 (95% CI 58.1–80.7) and 78.9 (95% CI 65.7–92.2) at 24 weeks. In the low‐dose arm, the mean score was 61.0 (95% CI 47.0–75.1) at baseline and 79.4 (95% CI 56.3–84.8) at 24 weeks. In the sham arm, the mean baseline score was 56.5 (95% CI 42.2–70.9) and 70.5 (95% CI 56.3–84.8) at 24 weeks. The greatest improvement in score was in the low‐dose arm (effect size 0.67) (Table A8).

#### 3.3.3 Wound‐Qol‐14

##### 3.3.3.1 Body Domain

The mean body score in the high‐dose ESWT arm (*n* = 25) at baseline was 1.12 (95% CI 0.67–1.57) and 0.39 (95% CI 0.13–0.65) at 24 weeks. In the low‐dose arm (*n* = 23), the mean score was 0.78 (95% CI 0.49–1.08) at baseline and 0.25 (95% CI 0.07–0.43) at 24 weeks. In the sham arm (*n* = 26), the mean score was 0.99 (95% CI 0.66–1.32) at baseline and 0.41 (95% CI 0.12–0.68) at 24 weeks. The low‐dose arm had the greatest improvement (effect size 3.0) (Table A9).

##### 3.3.3.2 Psych Domain

The mean psych score in the high‐dose ESWT arm was 1.69 (95% CI 1.17–2.21) at baseline and 1.08 (95% CI 0.51–1.64) at 24 weeks. In the low‐dose arm, the mean score was 1.79 (95% CI 1.21–2.37) at baseline and 0.76 (95% CI 0.16–1.36) at 24 weeks. In the sham arm, the mean score was 1.88 (95% CI 1.32–2.43) at baseline and 1.56 (95% CI 0.97–2.15) at 24 weeks. The greatest improvement was in the low‐dose arm (effect size 1.33) (Table A9).

##### 3.3.3.3 Everyday Life Domain

The mean everyday life score in the high dose ESWT arm at baseline was 1.66 (95% CI 1.10–2.23) and 0.79 (95% CI 0.27–1.31) at 24 weeks. In the low‐dose arm, the mean score at baseline was 1.68 (95% CI 1.12–2.24) and 0.78 (95% CI 0.21–1.34) at 24 weeks. In the sham arm, the mean score was 1.85 (95% CI 1.40–2.30) at baseline and 1.21 (95% CI 0.63–1.79) at 24 weeks. The greatest improvement was in the low‐dose arm (effect size 1.22) (Table A9).

##### 3.3.3.4 Global Score

The mean global score was 1.45 (95% CI 1.02–1.88) in the high‐dose ESWT arm at baseline and 0.74 (95% CI 0.34–1.15) at 24 weeks. In the low‐dose arm, the mean global score was 1.41 (95% CI 0.98–1.84) at baseline and 0.60 (95% CI 0.18–1.01) at 24 weeks. In the sham arm, the mean global score was 1.54 (95% CI 1.16–1.93) at baseline and 1.05 (95% CI 0.62–1.47) at 24 weeks. The low‐dose arm had the greatest improvement in the global score (effect size 1.53) (Table A9).

### 3.4 Adverse Events

#### 3.4.1 Side Effects

The most frequent side effect probably or definitely related to ESWT was tingling while the ESWT was being delivered, followed by pain and discomfort (Table 5). More side effects were reported by participants in the sham arm compared to participants in the high‐ and low‐dose ESWT arms.

**TABLE 5:** ESWT side effects.

Side effect, *n* (%)	High‐dose ESWT (*N* = 25)	Low‐dose ESWT (*N* = 23)	Sham ESWT (*N* = 26)
*ESWT Session 1*			
Burn	1 (4.0)	0	0
Pain	1 (4.0)	1 (4.3)	0
Discomfort	1 (4.0)	0	1 (3.8)
Pins and needles	1 (4.0)	0	1 (3.8)
Pressure	0	0	1 (3.8)
Awareness of a new sensation	0	0	1 (3.8)
Tingling	1 (4.0)	1 (4.3)	4 (15.4)
Local warmth	1 (4.0)	0	0
*ESWT Session 2*			
Pain	0	1 (4.3)	0
Skin pigmentation	0	0	1 (3.9)
Discomfort	0	0	0
Local warmth	0	1 (4.3)	0
Erythema	1 (4.0)	0	0
Increase in thread veins	0	0	1 (3.9)
Tingling	1 (4.0)	0	3 (11.5)
*ESWT Session 3*			
A quick pulse	0	1 (4.3)	0
Tapping	0	1 (4.3)	0
Tingling	1 (4.0)	0	0

#### 3.4.2 Serious Adverse Events

No serious adverse events were related to ESWT. During the pilot trial, 4 (15.4%) participants in the sham arm required revascularisation. Two (8.0%) participants in the high dose arm, two (8.7%) participants in the low dose arm and 2 (7.7%) participants in the sham arm required debridement/minor amputation in the operating theatre. Two (8.0%) participants in the high dose arm, one (4.3%) participant in the low dose arm and one (3.8%) participant in the sham arm underwent a major lower limb amputation. There were no deaths during the 24‐week follow‐up period.

### 3.5 Healthcare Resource Use

The mean number of consultant‐led outpatient appointments and podiatry appointments was higher in the sham arm than in the high and low dose arms. The mean number of practice nurse appointments was higher in the high dose arm. The mean number of district nurse visits and GP appointments was the same in the three arms (Table 6).

**TABLE 6:** Healthcare resource use.

Healthcare resource	Mean (95% CI)		
	High‐dose ESWT (*N* = 25)	Low‐dose ESWT (*N* = 23)	Sham ESWT (*N* = 26)
Outpatient appointments	5 (3–7)	4 (2–7)	6 (3–8)
GP appointments	1 (0–1)	1 (0–2)	1 (0–2)
Hospital admissions	1 (0–1)	1 (0–1)	1 (0–1)
Length of stay	3 (1–6)	5 (1–10)	3 (1–5)
Practice nurse appointments	6 (1–11)	3 (0–5)	4 (1–7)
District nurse appointments	5 (0–10)	5 (0–10)	5 (1–10)
Podiatry appointments	7 (6–9)	7 (6–8)	8 (7–9)

## 4 Discussion

This pilot trial demonstrates the proposed method to recruit, deliver ESWT and follow‐up patients in the definitive trial is feasible. Although the percentage of eligible patients was in the amber stop/go criteria, this likely reflects the population of people with neuropathic and neuro‐ischaemia DFUs. The lower eligible rate was compensated for by the high concordance with treatment and follow‐up [9, 38]. Of those excluded, the most frequent reason was insufficient arterial supply, who are likely to benefit more from revascularisation and patients with osteomyelitis, in which ESWT is unlikely to be effective. Overall, 15.6% of patients screened were eligible. This is comparable to other trials undertaken in similar healthcare settings [39, 40, 41].

Reasons eligible patients were not recruited including not being able to contact the patient, the patient not wanting to take part in research and no available transport. Despite using tactics to re‐contact patients who accepted a Patient Information Sheet, 10% (14/141) of eligible patients could not be re‐contacted [42]. The reasons for declining participation were explored in a qualitative study nested in the pilot trial and will be reported elsewhere. The use of placebos and expressing a treatment preference are known reasons that patients decline trial participation [43, 44]. The pilot RCT funded transport to research appointments and coordinated appointments with existing healthcare appointments. It is possible that the burden of additional appointments, rather than the transport, was the barrier to participation [45]. Reasons for non‐recruitment were comparable to other trials with a similar patient pool [39, 40]. The pilot RCT recruited 82.2% of the target sample size during the 18‐month recruitment period. This will guide the expected recruitment time frame in a definitive trial. Methods to increase recruitment, such as use of the Quintet Recruitment Intervention methods and monetary incentives, could be considered in a future trial [43, 46].

Attendance at the face‐to‐face follow‐up appointments decreased over time, with a greater reduction in the active ESWT arms. This could also explain some of the missing data. A hybrid approach with face‐to‐face reviews, video calls and a patient‐friendly platform to submit DFU photos may improve data collection during follow‐up. The use of patient‐submitted wound photos is increasingly popular and may have a place in trial follow‐up [47, 48, 49]. Extra support is likely needed for participants with less experience using technology [50]. The option to complete remote follow‐up for those who had healed may also increase trial retention. The pilot trial attrition at 6 months was only 12.2%, significantly less than the expected 30%.

Examining the missing data items, the quality‐of‐life questionnaires had the lowest completion. This could reflect the number of questionnaires participants were requested to complete. The EQ‐5D‐5L had slightly better completion at 24 weeks, compared to the disease‐specific quality‐of‐life questionnaires. More participants healed by this point and may have felt the disease‐specific quality‐of‐life questionnaires were not as relevant anymore. A future trial should select a disease‐specific and generic quality‐of‐life tool to reduce the burden of paperwork on trial participants.

In the pilot RCT participants were stratified by DFU size and lower limb perfusion to balance two important healing‐related factors and to test whether it resulted in overall balancing between the arms [51]. At baseline, differences remained between the arms, such as patient age, duration of diabetes and the DFU, DFU size and aetiology of DFU. There was also imbalance in quality‐of‐life scores, with participants in the sham arm reporting a better generic quality‐of‐life (EQ‐5D‐5L) and the high dose arm reporting a better disease‐specific quality‐of‐life (DFS‐SF). While these differences could be due to a small sample size, a further trial should consider using additional strata to balance for the other factors that affect healing, or use minimisation [52].

The results of this pilot RCT signal that ESWT and particularly high dose ESWT, is associated with accelerated healing compared to standard care alone. Reduction in healing time and DFU size with ESWT has been reported in other two‐arm RCTs [53, 54, 55, 56, 57, 58]. This supports the hypothesis that ESWT may improve wound healing through an increase in the expression of endothelial growth factor, vascular endothelial growth factor and endothelial nitrous oxide, leading to the proliferation of vascular endothelial cells, fibroblasts and modulation of interleukins [16, 17, 18, 19]. The clinical outcomes presented here require exploration in a definitive trial to evaluate whether differences are significant. Positive ulcer‐related outcomes in the high dose arm did not appear to always correlate with improved quality of life. Further sufficiently powered trials are required to explore whether this is due to underpowering or lack of sensitivity of DFU disease‐specific quality of life tools, which has previously been proposed [59].

The pilot trial has limitations. During the trial follow‐up, the incidence of DFU infection between follow‐up visits was not collected. This could have explained some of the variation in DFU size and quality‐of‐life scores between the arms. The study was conducted in a single tertiary care hospital in England and therefore results may not translate into different healthcare settings. The demographics of the pilot RCT participants were homogenous and may not represent diversity in other healthcare settings. This could mean that trial processes may not function as expected in settings with fewer links to the local community podiatry services, less research resources and greater ethnic diversity in the local population. Monitoring during a multi‐centre internal pilot trial would help to explore whether the pilot outcomes presented here translate into the different healthcare settings [60].

This pilot trial supports the development of a definitive trial exploring the dosing of ESWT for DFU healing. Results of the pilot trial have defined expected eligibility and recruitment rates and identified potential problems with trial follow‐up. This will allow for the trial design and processes to be amended prior to undertaking a definite trial exploring the efficacy of different doses of ESWT in augmenting DFU healing.

## Ethics Statement

The study was approved by the NHS Health Research Authority (IRAS: 311664, REC reference: 22/WA/0089) and sponsored by the Hull University Teaching Hospitals NHS Trust. The study was undertaken in line with the Declaration of Helsinki 1975 [61].

## Consent

All participants provided written informed consent before participation.

## Conflicts of Interest

The authors declare no conflicts of interest.

## Supporting information


**Data S1:** Supporting Information.

## Data Availability

The data that support the findings of this study are available from the corresponding author upon reasonable request.

## Appendix A

### Pilot Outcomes

**TABLE A1:** Reasons screened patients were ineligible for trial participation.

Reason for ineligibility	Number of patients (*n*)	Percentage
Wound less than 4 weeks old	274	35.9
ABPI less than 0.7 or absolute toe pressure less than 50 mmHg	108	14.2
Osteomyelitis	108	14.2
Anticoagulation	80	10.5
Interdigital ulcer	46	6.0
Cancer	35	4.6
Non‐compliance	30	3.9
Admitted for amputation	27	3.5
Lacks capacity	17	2.2
Participant in another wound related trial	15	2.0
Unable to apply ESWT	13	1.7
Ulcer above the malleolus	2	0.3
Does not speak English	4	0.5
Palliative	2	0.3
Does not have diabetes	1	0.1
Other	1	0.1

**TABLE A2:** Reasons eligible patients declined to participate.

Reason for not recruiting	Number of patients (*n*)	Percentage
Research team unable to re‐contact with the patient	14	20.9
Patient declined participation	13	19.4
Patient reported transport problems to get to hospital	8	11.9
DFU healed at baseline visit	6	9.0
Patient prefers standard care	4	6.0
Patient is not willing to make the time commitment to participate	4	6.0
Patient did not want to leave the dog three times a week	2	3.0
Patient was ‘fed up’ of hospitals	2	3.0
Patient felt the DFU was almost healed so did not need ESWT	2	3.0
Patient reports having too many existing appointments already	2	3.0
Patient thinks there are too many ESWT visits	2	3.0
Patient is unable to get time off work to attend ESWT sessions	2	3.0
Patient did not agree with placebos	1	1.5
Patient felt too frail to take part	1	1.5
Patient thinks there are too many research visits	1	1.5
Patient felt too tired to do research	1	1.5
Patient felt too unwell to do research	1	1.5
Patient felt too upset to talk about the DFU	1	1.5

### Standard of Care (See Tables A3, A4, A5, A6)

**TABLE A3:** Dressings used over the trial period.

Dressing type	Participants,^a^ *n* (%)
Soft polymer with absorbent pad	71 (95.6)
Crepe (± wool)	14 (18.9)
Povidone–iodine fabric dressing	35 (47.3)
Polyurethane foam film dressing	30 (40.5)
Anti‐microbial hydrocolloid fibrous dressing	30 (40.5)
Hydrocolloid‐fibrous dressing	15 (20.3)
Protease modulating dressings	6 (8.1)
Alginate with silver	1 (1.4)
Antimicrobial with foam dressing	1 (1.4)
Other antimicrobial dressing^b^	9 (12.2)

^a^ Number of patients who received the type of dressing at least once during the trial period.

^b^ Low adherence with silver, Calgitrol paste, Cutimed Sorbact.

**TABLE A4:** Offloading devices used over the trial period.

Offloading method	Participants,^a^ *n* (%)
Offloading sandals (heel/forefoot)	38 (51.4)
Felt padding	25 (33.8)
Custom total contact insoles	22 (29.7)
Custom orthotic footwear	20 (27.0)
Removable heel cast	8 (10.8)
Below ankle removable total contact cast	7 (9.5)
Midfoot removable total contact cast	1 (1.4)
Removable below knee offloading device	1 (1.4)
Wheelchair	11 (14.9)
Gait aid	15 (20.3)
Heel protector	2 (2.7)

^a^ Number of patients who received the type of offloading at least once during the trial period.

**TABLE A5:** Debridement^a^ during the trial period.

Time point	Participants, *n* (%)
Baseline	39/74 (52.7)
ESWT Session 3	37/74 (50.0)
6 weeks follow‐up	45/55 (81.2)
12 weeks follow‐up	34/45 (75.5)
24 weeks follow‐up	29/29 (100)

^a^ If required (unhealed DFU). In between trial appointments, participants underwent sharp debridement if required at the routine podiatry clinic appointments.

**TABLE A6:** Antibiotics used to treat DFU infection.

Antibiotic, *n* (%)	High dose ESWT	Low‐dose ESWT	Sham ESWT
*Baseline*			
Doxycycline	1 (4.0)	4 (17.4)	6 (23.1)
Cotrimoxazole	2 (8.0)	0	1 (3.8)
Flucloxacillin	0	0	1 (3.8)
Clarithromycin	1 (4.0)	0	0
Moxifloxacin	1 (4.0)	0	0
*ESWT Session 3*			
Doxycycline	2 (8.0)	3 (13.0)	6 (23.1)
Cotrimoxazole	0	0	1 (3.8)
Metronidazole	0	0	1 (3.8)
Clarithromycin	1 (4.0)	0	0
Moxifloxacin	1 (4.0)	0	0
*6 weeks follow‐up*			
Doxycycline	3 (12.0)	0	3 (11.5)
Cotrimoxazole	2 (8.0)	2 (8.7)	3 (11.5)
Flucloxacillin	1 (4.0)	3 (13.0)	2 (7.7)
Metronidazole	1 (4.0)	0	0
Co‐amoxiclav	1 (4.0)	0	0
*12 weeks follow‐up*			
Doxycycline	2 (8.0)	2 (8.7)	1 (3.8)
Cotrimoxazole	1 (4.0)	0	1 (3.8)
Flucloxacillin	1 (4.0)	0	0
Linezolid	0	0	1 (3.8)
Ertapenem and meropenem beads	0	1 (4.3)	0
Vancomycin and gentamicin beads	0	0	1 (3.8)
*24 weeks follow‐up*			
Doxycycline	2 (8.0)	3 (13.0)	2 (7.7)
Cotrimoxazole	2 (8.0)	1 (4.3)	2 (7.7)
Flucloxacillin	1 (4.0)	0	0
Linezolid	0	0	1 (3.8)

### Quality‐of‐Life (See Tables A7 and A8)

**TABLE A7:** EQ‐5D‐5L effect sizes between baseline and each follow‐up point.

Domain	Effect size		
	High dose	Low dose	Sham
*ESWT Session 3*			
UV	0.01	0.12	−0.01
VAS	0.18	−0.01	−0.06
*6‐week follow‐up*			
UV	0.08	0.09	0.54
VAS	0.25	0.18	0.67
*12‐week follow‐up*			
UV	0.11	0.27	−0.03
VAS	0.25	−0.06	0.20
*24‐week follow‐up*			
UV	0.12	0.34	0.06
VAS	0.20	−0.04	0.06

Abbreviations: ESWT: extracorporeal shockwave therapy; UV: utility value; VAS: visual analogue scale.

**TABLE A8:** DFS‐SF effect sizes between baseline and each follow‐up point.

Domain	Effect size		
	High dose	Low dose	Sham ESWT
*ESWT Session 3*			
Leisure	0.24	−0.24	0.11
Physical health	0.16	−0.43	0.15
Dependence	0.10	−0.09	0.06
Negative emotions	0.24	0	0.09
Worried about ulcers/feet	0.10	−0.08	0.13
Bothered by ulcer care	0.29	0.16	0.17
*6 week follow‐up*			
Leisure	0.17	0	−0.08
Physical health	0.24	0.32	0.33
Dependence	−0.11	−0.42	−0.10
Negative emotions	−0.00	−0.15	−0.21
Worried about ulcers/feet	0.09	−0.09	0.09
Bothered by ulcer care	0.09	0.12	0.11
*12‐week follow‐up*			
Leisure	0.54	0.29	0.11
Physical health	0.40	0	0.28
Dependence	0.13	0.43	0.22
Negative emotions	0.25	0.17	−0.06
Worried about ulcers/feet	0.30	0.18	0.20
Bothered by ulcer care	0.43	0.80	0.06
*24‐week follow‐up*			
Leisure	0.78	0.44	0.54
Physical health	0.51	0.14	0.26
Dependence	0.42	0.50	0.33
Negative emotions	0.24	0.40	0.21
Worried about ulcers/feet	0.48	0.28	−0.05
Bothered by ulcer care	0.47	0.67	0.25

**TABLE A9:** Wound‐Qol‐14 effect sizes between baseline and each follow‐up point.

Domain	Effect size		
	High dose	Low dose	Sham ESWT
*ESWT Session 3*			
Body	0.14	0.67	0.33
Psyche	0.32	0.09	0.15
Everyday life	0.15	0	0.16
Global	0.17	0.36	0.10
*6‐week follow‐up*			
Body	−0.50	−1.32	−0.50
Psyche	−0.12	−0.20	−0.40
Everyday life	−0.30	−0.32	−0.26
Global	−0.62	−0.57	−0.60
*12‐week follow‐up*			
Body	1.00	2.00	0.93
Psyche	0.57	0.75	0.18
Everyday life	0.88	0.63	0.14
Global	0.37	1.39	0.25
*24‐week follow‐up*			
Body	1.09	3.00	1.00
Psyche	0.44	1.33	−0.04
Everyday life	0.88	1.20	0.38
Global	0.67	1.53	0.31
